# Hospital Discharge Data for Guillain-Barré Syndrome and Influenza A (H1N1) Vaccine Adverse Events

**DOI:** 10.3201/eid1609.091837

**Published:** 2010-09

**Authors:** Timothy F. Jones, Marcy McMillian, Effie Boothe, Samir Hanna, L. Amanda Ingram

**Affiliations:** Author affiliation: Tennessee Department of Health, Nashville, Tennessee, USA

**Keywords:** Guillain-Barre syndrome, surveillance, adverse events, H1N1 vaccination, influenza, letter

**To the Editor:** As part of the public health response to the current pandemic (H1N1) 2009, surveillance for adverse events following vaccination for influenza A (H1N1) is a high priority (*1*). Surveillance for Guillain-Barré syndrome (GBS) has been of particular interest, because the syndrome was associated with the 1976–1977 swine influenza vaccine (*1**,**2*). To study this association, reliable ascertainment of recent incident cases of GBS is necessary.

GBS is an acute, immune-mediated paralytic disorder of the peripheral nervous system (*3*–*5*) with an estimated annual incidence of 0.8–1.9/100,000 (*6*). Most cases are associated with an antecedent infection (*6*). Several surveillance systems are in place to monitor rates of postvaccination GBS (*1*–*3*), most of which include a component of electronic administrative record review for case detection. Analysis of computerized medical databases is a well-established method of monitoring for vaccine adverse events (*7*). Although the validity of such data varies, depending on the diagnosis and region, few studies have evaluated the use of hospital discharge data for GBS specifically (*8**,**9*).

We reviewed the Tennessee Department of Health Uniform Hospital Discharge Dataset for all hospital discharge diagnoses in 4 major metropolitan regions of Tennessee in 2002–2003 with codes from the International Classification of Diseases, 9th Revision, Clinical Modification (ICD-9-CM), that might indicate acute GBS. Records with ICD-9-CM code 357.0 (acute infective polyneuritis) or other combinations suggestive of GBS within the top 10 diagnoses were requested. These data were compared with information on cases identified by directly requesting lists of patients with discharge diagnoses of GBS from hospital medical record departments. Charts of all reported cases were validated by chart review. Patients were classified as having acute GBS if they met Brighton Criteria Levels 1, 2, or 3 (*10*).

A total of 344 records of possible cases of acute GBS were identified. Of these cases, 215 (63%) were identified through the state hospital discharge database, 315 (92%) were reported directly by hospitals, and 186 (54%) were identified by both systems. Among all suspected cases identified, only 103 (30%) met criteria for acute GBS (annual rate 2.1/100,000 population), 14 (4%) were out-of-state residents, 114 (33%) were nonacute cases that occurred before the study period and patients were readmitted for other reasons, 90 (26%) had no documentation of GBS in the medical record, 17 (5%) were duplicate reports, and 6 (2%) had insufficient information for further investigation. The predictive-value positive of a GBS diagnostic code from the statewide hospital discharge database representing acute GBS was only 30%. Of the 103 confirmed cases, 26 (25%) would have been missed if only the state hospital discharge database was used to identify potential cases.

Of 103 cases, all were identified with ICD-9-CM diagnosis code 357.0; in 91 (88%) cases, this was the primary diagnosis. Other combinations of codes did not identify additional cases. Of cases of acute GBS identified, 32 (30%) met only clinical criteria (Brighton Level 1), 40 (39%) had either laboratory or electrophysiologic evidence (Brighton Level 2), and 32 (31%) had both (Brighton Level 3).

Because the 2 surveillance systems we compared both relied on medical record discharge diagnoses, they were not independent, and we could not perform a capture/recapture analysis. Because GBS is a diagnosis for which the great majority of patients are hospitalized, and our overall incidence rate is within the range identified in other studies, it is likely that the combination of these methods is reasonably sensitive. The administrative hospital discharge database could not be relied on to confirm that all coded GBS cases were acute. Even if the 114 nonacute cases could easily have been identified and excluded from the initial list of 344 records, only 103 (45%) of the remaining 230 reports were identified as confirmed acute cases.

Although the use of large hospital discharge databases may be useful as an adjunct for identification of GBS cases as part of public health surveillance, they lack sufficient sensitivity or specificity to be relied upon exclusively. The poor specificity of the system is particularly problematic for public health surveillance. A large investment of time and resources was necessary to perform manual chart reviews to confirm possible cases, two-thirds of which were ultimately found not to be cases at all. Statewide administrative hospital discharge diagnosis databases should not be solely relied on for GBS surveillance. Additional methods of reliable and efficient ascertainment and verification of cases are crucial to ensure valid data. Obtaining reliable methods is particularly important for urgent situations such as current surveillance for adverse events after pandemic (H1N1) 2009 virus vaccination, in which the detection of problems will have immediate public health effects.
